# Effectiveness of the Oral Human Attenuated Rotavirus Vaccine: A Systematic Review and Meta-analysis—2006–2016

**DOI:** 10.1093/ofid/ofy292

**Published:** 2018-11-12

**Authors:** Corinne Willame, Marije Vonk Noordegraaf-Schouten, Emilia Gvozdenović, Katrin Kochems, Anouk Oordt-Speets, Nicolas Praet, Rosa van Hoorn, Dominique Rosillon

**Affiliations:** 1 GSK, Wavre, Belgium; 2 Pallas Health Research and Consultancy, Rotterdam, the Netherlands

**Keywords:** effectiveness, meta-analysis, *Rotarix*, rotavirus, systematic literature review

## Abstract

**Background:**

Gastroenteritis caused by rotavirus accounts for considerable morbidity in young children. We aimed to assess the vaccine effectiveness (VE) of the oral rotavirus vaccine *Rotarix*, as measured by laboratory-confirmed rotavirus infection after referral to hospital and/or emergency departments in children aged <5 years with gastroenteritis.

**Methods:**

We performed a systematic search for peer-reviewed studies conducted in real-life settings published between 2006 and 2016 and a meta-analysis to calculate the overall *Rotarix* VE, which was further discriminated through stratified analyses.

**Results:**

The overall VE estimate was 69% (95% confidence interval [CI], 62% to 75%); stratified analyses revealed a non-negligible impact of factors such as study design and socioeconomic status. Depending on the control group, VE ranged from 63% (95% CI, 52% to 72%) to 81% (95% CI, 69% to 88%) for unmatched and matched rotavirus test–negative controls. VE varied with socioeconomic status: 81% (95% CI, 74% to 86%) in high-income countries, 54% (95% CI, 39% to 65%) in upper-middle-income countries, and 63% (95% CI, 50% to 72%) in lower-middle-income countries. Age, rotavirus strain, and disease severity were also shown to impact VE, but to a lesser extent.

**Conclusions:**

This meta-analysis of real-world studies showed that *Rotarix* is effective in helping to prevent hospitalizations and/or emergency department visits due to rotavirus infection.

Rotavirus (RV) is the major cause of severe gastroenteritis (GE) diseases, which amount to a considerable burden of disease in children younger than age 5 years [1]. Although a global decline in mortality was observed in the last decades, RV diseases still accounted for an estimated 215 000 deaths in this age group in 2013 [2].

Vaccination is the best preventive approach against RV diseases [1]. The oral live-attenuated human RV vaccine *Rotarix* (GSK, Belgium) was introduced in routine immunization programs as of 2006. In 2009, the World Health Organization recommended the global implementation of RV vaccination in infants [3]. By June 2017, 85 countries had introduced RV vaccination in their national immunization program (NIP), with an additional 7 countries including it in subnational programs and other countries making the vaccines available for private market use [4, 5].

*Rotarix* is a 2-dose-shedule oral live-attenuated human RV vaccine, recommended for active immunization against GE due to RV infection (RVGE) in infants aged 6–24 weeks [6]. The 2 doses of *Rotarix* should be administered at least 4 weeks apart, and the vaccination course must be completed by 24 weeks of age [6]. *Rotarix* was shown to have a favorable benefit/risk profile in infants and was efficacious against severe GE- or RV-associated hospitalization in several large clinical trials conducted worldwide [7–14], with a vaccine efficacy of 85%–96% demonstrated against these end points [13, 14]. Favorable data from clinical trials were further supported by postlicensure studies conducted over a period of more than 10 years since the introduction of the vaccine in routine immunization programs [5]. *Rotarix* was shown to provide broad protection against severe RVGE caused by nonvaccine RV strains; that is, efficacy or effectiveness has been demonstrated against 9 different strains [6].

This study evaluated the vaccine effectiveness (VE) of *Rotarix*, as measured by laboratory-confirmed RV infection after referral to hospitals and/or the emergency department (ED), in children with GE diseases in real-world settings. We conducted a systematic literature review and a meta-analysis of the VE of 2- or 1-dose *Rotarix* vaccination data published between January 1, 2006, and July 7, 2016.

Figure 1 represents a “Focus on the Patient Section,” which elaborates on the clinical relevance and impact of the study, to be shared with patients by health care professionals.

**Figure 1.**
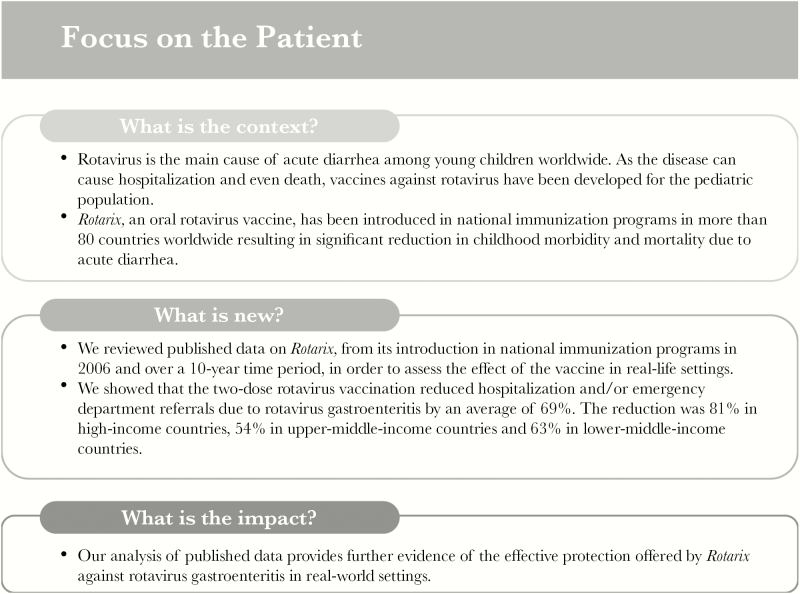
Focus on the patient section.

## METHODS

### Systematic Literature Review

We performed a systematic search of the PubMed and Cochrane databases for peer-reviewed articles published from January 1, 2006, to July 7, 2016, using prespecified terms related to RV vaccines, as detailed in the Supplementary Data. We included papers in any language reporting data from postlicensure or original studies assessing the VE of *Rotarix* (full inclusion and exclusion criteria are presented in the Supplementary Data).

Relevant references were selected by a 3-step selection procedure. First, titles and abstracts identified through the search were screened based on their relevance to the objectives, with a random sample of 30% of titles and abstracts being screened in duplicate. Second, a full-text review of articles selected during the first step was performed, with the first 10% of the articles being appraised by 2 reviewers. Third, further scrutiny of the articles during the data extraction phase was applied. For example, when 2 included articles described results of the same study, we only included 1 of the articles in the meta-analysis to avoid double inclusion of data (ie, the article published most recently or with the most relevant data). In addition, the reference list of meta-analyses or systematic reviews was checked for relevant articles that could have been missed. The quality of the selected articles was assessed using the Coordination of Cancer Clinical Practice Guidelines (CoCanCPG) [15].

We extracted and summarized the following data as a minimum: study design, setting and period, study objectives, study country and its socioeconomic status (SES; according to the World Bank list of economies classification [16]), type of control group used (matched/unmatched hospital, test-negative, community/neighborhood controls), clinical setting (hospitalizations or ED visits), RV strain type (homotypic, fully/partly heterotypic), disease severity (mild, moderate, severe, and very severe, according to the Vesikari score list [17]), reports on vaccine introduction in the NIP, and vaccination coverage, when available.

### Meta-analyses

A meta-analysis was performed to assess the overall VE of *Rotarix*, as measured by laboratory-confirmed RV infection after referral to hospitals and/or EDs in children with GE under 5 years of age, as reported by observational studies identified by the systematic literature search (see the Supplementary Data for a full list of inclusion criteria).

We estimated the overall VE in children receiving 2 doses of *Rotarix* (main analysis). The secondary objectives of the meta-analysis were to assess the VE according to the number of *Rotarix* doses provided (1 or 2), type of controls used in the studies, the SES of the country, RV strain type, age (<1 or ≥1 year), and disease severity. Stratification analyses were carried out on 1 level (age, RV strain, SES, and disease severity) for 1 *Rotarix* dose and the complete schedule, whereas 2-level stratified analyses were conducted only for 2 doses of *Rotarix* (by age and SES and by strain and SES). A meta-analysis was performed only when at least 4 VE estimates could be included. To investigate the effect of specific study parameters on the VE, sensitivity analyses were conducted by excluding studies reporting on ED referrals only or primary health care centers, high-income countries and unmatched control groups, or unadjusted data.

### Statistical Methods

In performing the analyses, several considerations were predefined for each included study. If VE estimates were presented for multiple control groups, only 1 estimate was selected as follows: hospital controls were preferred above community controls; in case of multiple hospital controls, matched controls were used; and when nonmatched control groups were studied, test-negative controls were preferred. When both crude and covariate-adjusted VE were provided in studies, adjusted estimates were used for the meta-analysis.

VE was defined as the percent reduction in the odds of referral to hospitals and/or EDs due to RVGE disease among vaccinated children compared with unvaccinated children. Meta-analyses were performed on odds ratios (ORs) and 95% confidence intervals (CIs); log(OR) and standard error of log(OR) were computed. If the ORs and 95% CIs were not available in the included studies, these were calculated from the estimates included in the articles using the formula OR = 1 – (VE/100) [18].

The random-effect model (using the DerSimonian-Laird approach) [19] was used for the main model, but the fixed-effect model (using the inverse variance method) [20] was also employed to calculate pooled ORs. The level of study heterogeneity was assessed by computing the Higgins *I*^2^ test, along with visual assessment of the funnel plots [21]. *I*^2^ values of <25%, 25%–50%, 50%–75%, and >75% were considered very low, low, medium, and high heterogeneity, respectively [22].

Publication bias was investigated for the overall 1- and 2-dose VE analyses, by visual assessment of funnel plots, and by Egger’s weighted regression test, with a 2-sided *P* value of <.10 considered significant [23].

All analyses were performed without any adjustment for multiplicity using STATA v13.1.

## RESULTS

### Systematic Literature Review

**Figure 2.**
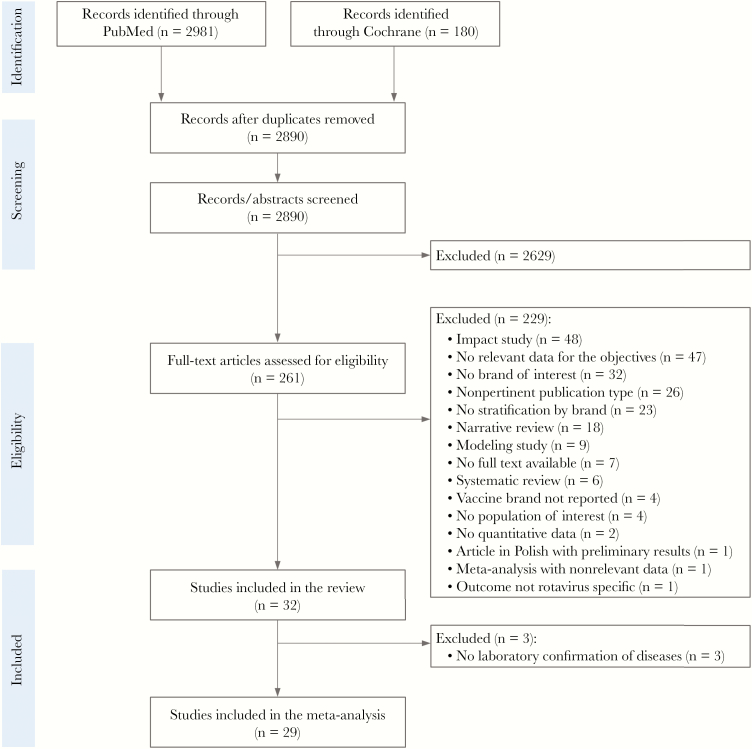
PRISMA flowchart.

After removal of duplicates, the search strategy yielded 2890 unique records. Following the screening of titles and abstracts, we retained 261 articles for full-text review, from which 32 studies [24–55] were identified as relevant for the assessed outcomes. Figure 2 gives a schematic overview of the selection procedure used. Study characteristics are summarized in Table 1.

**Table 1.** Characteristics of Studies Identified Through the Systematic Search

	Country/Region (SES)	NIP Introduction^a^ VC, %	Study Setting	Outcome	Study Design	Study Period	Age at Study Enrollment	Case confirmation
Studies included in the meta-analysis								
Armah et al. 2016 [24]	Ghana (LMIC)	NIP since 2012 VC: >90%	Hospital and ED	AGE RV	Case-control (test-negative)	Jan 2013–Dec 2014	6wk–5y	EIA
Bar-Zeev et al. 2015 [26]	Malawi (LIC)	NIP since Oct 2012 VC: 74.6% (2013), 92.4% (2014), 95.1% (2015)	ED	RV AGE	Matched case-control (matched community control, unmatched for test-negative control)	Oct 2012–Jun 2014	≥6wk–<5y	ELISA
Bar-Zeev et al. 2016 [25]	Malawi (LIC)	NIP since Oct 2012 VC: 74.6% (2013), 92.4% (2014), 95.1% (2015)	Hospital and ED	Acute RV diarrhea	Case-control (test-negative)	Oct 2012–Jun 2015	<5y	EIA + PCR
Beres et al. 2016 [27]	Zambia (LMIC)	NIP since 2013 VC: 58%–70%	Hospital^b^	Moderate to severe RV diarrhea	Case-control (test-negative)	Jul 2012–Oct 2013	0–59mo	EIA
Braeckman et al. 2012 [41]	Belgium (HIC)	NIP since Oct 2006 VC: 90%	Hospital^b^	RV GE	Matched case-control (hospital control)	Feb 2008–Jun 2010	14wk–39mo	Rapid test
Castilla et al. 2012 [42]	Spain/Navarre (HIC)	NIP since Aug 2006 but suspended in Mar 2010 VC: NR	Health care database^c^	RV GE	Nested case-control (test-negative)	Jan 2008–Jun 2011	3–59mo	ICA
Chang et al. 2014 [47]	Taiwan (HIC)	Private market since Aug 2006 VC: 24%–28% in controls	Hospital^b^	RV AGE	Matched case-control (test-negative and hospital control)	May 2009–Apr 2011	8–35mo	EIA
Correia et al. 2010 [32]	Brazil (UMIC)	NIP since Mar 2006 VC: >50%	Hospital and ED	Severe RV diarrhea	Unmatched case-control (test-negative and hospital control)	Mar 2006–Sep 2008	6mo–5y	ELISA
Cortese et al. 2013 [48]	US (HIC)	NIP since 2008 VC: NR	Hospital and ED	RV AGE	Matched case-control (matched test-negative IIS restricted control; unmatched for test-negative control)	Jan 2010–Jun 2011	≥56d–23mo	EIA
Cotes-Cantillo et al. 2014 [33]	Colombia (UMIC)	NIP since Jan 2009 VC: 94%	ED	RV diarrhea	Case-control (unmatched test-negative)	Jan 2011–Jan 2013	≥8wk–4y	Rapid test + ELISA
de Palma et al. 2010 [34]	El Salvador (LMIC)	NIP since Oct 2006 VC estimated at 50%	Hospital^b^	RV diarrhea	Matched case-control (neighborhood control)	Jan 2007–Jun 2009	<5y	EIA
Doll et al. 2015 [49]	Canada/Quebec (HIC)	NIP since Nov 2011 VC: 0% (2012), 90% (2014)	Hospital and ED	Acute diarrhea RV	Matched case-control (test-negative optimal matching based upon symptom onset)	Feb 2012–May 2014	8wk–3y	EIA+PCR
Gastanaduy et al. 2016a [35]	Guatemala (LMIC)	NIP since July 2010 VC estimated at 50%	Hospital and ED	RV diarrhea	Matched case-control (hospital control matched and unmatched test-negative control)	Jan 2012–Aug 2013	2–46mo	EIA
Gastanaduy et al. 2016b [29]	Botswana (UMIC)	NIP since July 2012 VC estimated at 70%	Hospital and ED	RV diarrhea	Case-control (test-negative)	Jun 2013–Apr 2015	≥4mo–<5y	EIA
Gheorghita et al. 2016 [43]	Moldova (LMIC)	NIP since July 2012 VC: 20% (2012), 40% (2013)	Hospital and ED	Acute RV diarrhea	Case-control (test-negative)	Oct 2012–July 2013	6mo–5y	EIA
Groome et al. 2014 [28]	South Africa (UMIC)	NIP since Aug 2009 VC: 96%	Hospital^b^	Acute diarrhea	Case-control (not matched, test-negative, and hospital)	Apr 2010–Oct 2012	18wk–23mo	ELISA
Ichihara et al. 2014 [36]	Brazil (UMIC)	NIP since Mar 2006 VC estimated at 70%	Hospital^b^	Acute diarrhea	Matched case-control (hospital control)	July 2008–Aug 2011	4–24mo	EIA+PAGE
Immergluck et al. 2016 [50]	US, Georgia (HIC)	NIP since 2008 in the US, since 2009 in Georgia VC: NR	Hospital and ED	RV diarrhea care	Case-control (test-negative)	Jan 2013–Jun 2013	≥56d–4y	EIA
Justino et al. 2011 [37]	Brazil, Belem (UMIC)	NIP since Mar 2006 VC: estimated 52%–69%	Hospital^b^	Severe RV GE	Matched case-control (hospital and neighborhood control)	May 2008–May 2009	≥12wk–5y	ELISA
Marlow et al. 2015 [44]	Portugal (HIC)	Private market since 2006 VC: 30%	ED	RV AGE	Matched case-control (test-negative)	Jan 2007–Jun 2012	8wk–36mo	Rapid test
Matthijnssen et al. 2014 [45]	Belgium (HIC)	NIP since Oct 2006 VC: 90%	Hospital^b^	RV GE	Matched case-control (hospital control)	Feb 2008–Jun 2010	14wk–39mo	PCR
Patel et al. 2013 [38]	Bolivia (LMIC)	NIP since Aug 2008 VC: 80%	Hospital and ED	RV diarrhea	Matched case-control (matched hospital control and unmatched for test-negative control)	Mar 2010–Jun 2011	8wk–36mo	ELISA
Payne et al. 2013 [51]	US (HIC)	NIP since 2008 VC: low	Hospital and ED; separate estimates available	RV AGE	Matched case-control (test-negative)	Nov 2009–Jun 2011	<5y	EIA
Payne et al. 2015 [52]	US (HIC)	NIP since 2008 VC: low	Hospital and ED; separate estimates available	RV AGE	Case-control (test-negative)	Dec 2011–Nov 2013	≥8mo–8y^d^	EIA+PCR
Pringle et al. 2016 [39]	Bolivia (LMIC)	NIP since Aug 2008 VC: NR	Hospital and ED	RV AGE	Case-control (test-negative)	Apr 2013–Mar 2014	2–59mo	EIA
Sahakyan et al. 2016 [46]	Armenia (LMIC)	NIP since Nov 2012 VC: 16% (Jan 2013), 57% (Jan 2013), 77% (Jan 2015)	Hospital^b^	RV AGE	Case-control (test-negative)	Nov 2012–Jun 2015	0–59mo	EIA
Snelling et al. 2009 [30]	Central Australia (HIC)	NIP since late 2006 VC: partial/low	Hospital^b^	AGE	Matched case-control (community control)	Mar 2007–Jul 2007	10wk–5y	Immunoassay
Snelling et al. 2011 [31]	Central Australia (HIC)	NIP since late 2006 VC: partial/low	Hospital^b^	GE	Nested and matched case-control (matched risk-cohort control; unmatched hospital control)	Sep 2008–Jun 2009	6wk–36mo	ICA
Yen et al. 2011 [40]	Mexico (UMIC)	NIP since 2006–2007 VC: 70% (age <5 y)	Hospital^b^	Severe GE	Matched case-control (community control)	Mar 2010–May 2010	15d–<2y	RNA extraction, electrophoresis
Studies excluded from the meta-analysis								
Gosselin et al. 2016 [53]	Canada/Quebec (HIC)	NIP since 2011 VC: 13.6% (Jan 2012), 85.9% (2014)	Health care database^c^	RV AGE and RVGE	Retrospective cohort (3 cohorts: vaccinated, unvaccinated, historical vaccinated [Aug 2008–Dec 2010])	Aug 2011–Dec 2013	<3y	ICD-10 diagnosis
Muhsen et al. 2010 [54]	Macabi, Israel (HIC)	Private market since 2007 VC: 55%	Health care database^c^	RV AGE	Incidence rate ratio (incidence rate in vaccinated individuals/incidence rate in nonvaccinated individuals)	Sep 2008–Jan 2009	<12mo	ICD-9 diagnosis
Perez-Vilar et al. 2015 [55]	Spain, Navarre region (HIC)	NIP since Aug 2006, suspended in Mar 2010	Health care database^c^	RV disease	Retrospective cohort	Jan 2007–Jun 2012	<3y	ICD-10 diagnosis

Abbreviations: AGE, acute gastroenteritis; ED, emergency department; EIA, enzyme immunoassay; ELISA, enzyme-linked immunosorbent assay; GE, gastroenteritis; HIC, high-income country; ICA, immuno-chromatographic assay; ICD-9, International Classification of Diseases, 9th Revision; ICD-10, ICD, 10th Revision; IIS, immunization information system; LIC, low-income country; LMIC, lower-middle-income country; NIP, national immunization program; NR, not reported; PAGE, polyacrylamide gel electrophoresis; PCR, polymerase chain reaction; RNA, ribonucleic acid; RV, rotavirus; SES, socioeconomic status; UMIC, upper middle-income country; VC, vaccination coverage.

^a^For countries where *Rotarix* was not included in the NIP, the date of its introduction on the private market was provided.

^b^Not clear if emergency departments were included.

^c^Includes hospital, ED setting, and primary health care centers.

^d^Estimates of vaccine effectiveness for *Rotarix* were obtained in children ≤62 months of age.

### Meta-analysis

The meta-analysis included 29 studies (Figure 2). For the 3 studies excluded [53–55], RV disease was only confirmed based on International Classification of Diseases codes and/or electronic medical records and was not based on laboratory results (Table 1).

### Characteristics of Selected Studies

Among the 29 studies included in the meta-analysis, 6 were conducted in African countries [24–29], 2 in Central Australia [30, 31], 9 in countries or regions from Latin America [32–40], 6 in Europe [41–46], 1 in Asia [47], and 5 in North America [48–52]. Most of them (27) were retrospective case-control studies conducted in hospital settings, and 2 were prospective case-control studies using electronic medical records from health care facilities [29, 41]. In all 29 studies, RV diseases requiring hospitalization or ED visits were assessed using robust laboratory testing to confirm the RV disease status. Based on the World Bank classification of economies, 2 studies were conducted in low-income countries [25, 26], 15 in lower and upper-middle-income countries, and 12 in high-income countries. Ten of the 29 studies presented results for more than 1 virus strain (Table 1).

All 29 studies included in the meta-analysis were case-control studies and fulfilled the quality criteria of the CoCanCPG checklist.

### Meta-analysis of the Effectiveness of *Rotarix* Vaccine

**Figure 3.**
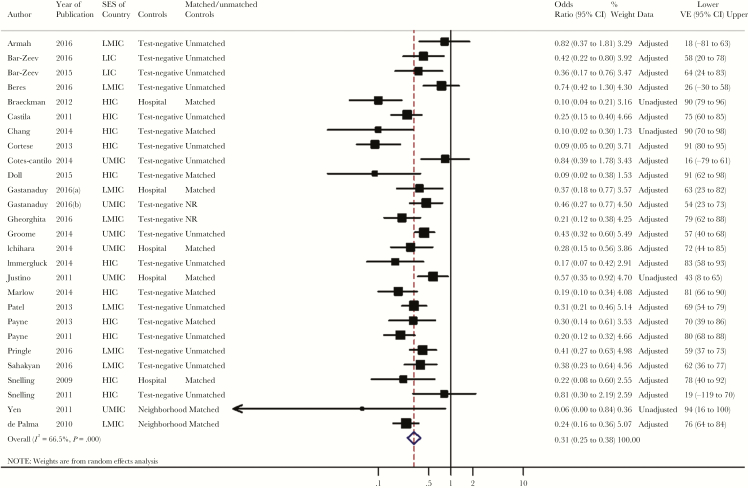
Estimated pooled vaccine effectiveness for 2 doses of *Rotarix* against laboratory-confirmed rotavirus infection after hospital and/or emergency department visits. Abbreviations: CI, confidence interval; HIC, high-income country; LIC, low-income country; LMIC, lower-middle-income country; NR, not reported; SES, socioeconomic status; UMIC, upper-middle-income country; VE, vaccine effectiveness.

The main analysis included 27 studies evaluating the overall VE of 2 doses of *Rotarix*. Two [32, 45] of the 29 studies identified by the systematic search were excluded from the main analysis, as only stratified data were presented without reporting overall results. The reported or calculated ORs per study ranged from 0.06 to 0.84. The overall VE estimate was 69% (95% CI, 62% to 75%), with medium heterogeneity (*I*^2^ = 67%; 95% CI, 50% to 78%) observed between studies (Table 2, Figure 3). Among the 12 studies included in the secondary analysis for 1 dose of *Rotarix*, the pooled VE was 46% (95% CI, 34% to 57%) with an *I*^2^ of 34% (95% CI, 0% to 67%), showing low between-study heterogeneity (Supplementary Figure 1).

**Table 2.** Overview of Overall Odds Ratios and Vaccine Effectiveness Against Laboratory-Confirmed Rotavirus Infection After Hospital and/or Emergency Department Visits for *Rotarix* Vaccination Resulting From Meta-analysis^a^

Analyses	No.	OR (95% CI)		*I* ^2^ (95% CI)	*P* Value (Cochrane Q-Test)	VE (95% CI)^b^
RE Model	FE Model
Primary analysis						
2 doses	27	0.31 (0.25–0.38)	0.33 (0.29–0.37)	67 (50–78)	<.001	69 (62–75)
1 dose	12	0.54 (0.43–0.66)	0.57 (0.49–0.66)	34 (0–67)	.115	46 (34–57)
Secondary analyses, 2 doses						
Hospital controls, 2 doses, matched	9	0.24 (0.15–0.36)	0.26 (0.22–0.35)	62 (22–82)	.007	76 (64–85)
Test-negative matched controls	4	0.19 (0.12–0.31)	0.20 (0.13–0.30)	10 (0–86)	.345	81 (69–88)
Other hospital matched controls	5	0.28 (0.16–0.51)	0.28 (0.22–0.36)	71 (27–89)	.008	72 (49–84)
Hospital controls, 2 doses, unmatched	14	0.37 (0.28–0.48)	0.36 (0.31–0.42)	72 (51–83)	<.001	63 (52–72)
Test-negative unmatched controls	14	0.37 (0.28–0.48)	0.36 (0.31–0.42)	72 (51–83)	<.001	63 (52–72)
Neighborhood/community controls, 2 doses	5	0.32 (0.20–0.49)	0.29 (0.22–0.39)	42 (0–79)	.140	68 (51–80)
Matched controls	5	0.32 (0.20–0.49)	0.29 (0.22–0.39)	42 (0–79)	.140	68 (51–80)
Stratified analyses						
2 doses, LMIC	8	0.37 (0.28–0.50)	0.35 (0.30–0.42)	62 (18–82)	.010	63 (50–72)
2 doses, UMIC	6	0.46 (0.35–0.61)	0.46 (0.37–0.57)	28 (0–70)	.225	54 (39–65)
2 doses, HIC	11	0.19 (0.14–0.26)	0.19 (0.16–0.24)	49 (0–75)	.033	81 (74–86)
1 dose, LMIC	5	0.63 (0.52–0.77)	0.63 (0.52–0.77)	0 (0–79)	.454	37 (23–48)
1 dose, UMIC	4	0.52 (0.40–0.67)	0.52 (0.40–0.67)	0 (0–85)	.506	48 (33–60)
2 doses, high severity	14	0.36 (0.26–0.50)	0.35 (0.29–0.42)	67 (41–81)	<.001	64 (50–74)
2 doses, very high severity	9	0.40 (0.26–0.62)	0.37 (0.26–0.53)	30 (0–68)	.177	60 (38–74)
1 dose, high severity	6	0.62 (0.46–0.84)	0.62 (0.46–0.84)	0 (0–75)	.577	38 (16–54)
1 dose, very high severity	4	0.70 (0.38–1.28)	0.73 (0.43–1.23)	20 (0–88)	.289	30 (-28–62)
2 doses, homotypic strain	5	0.11 (0.07–0.18)	0.11 (0.07–0.18)	0 (0–79)	.728	89 (82–93)
2 doses, partly heterotypic strain	12	0.28 (0.22–0.35)	0.28 (0.22–0.35)	0 (0–58)	.902	72 (65–78)
2 doses, fully heterotypic strains	15	0.35 (0.26–0.46)	0.38 (0.23–0.45)	53 (16–74)	.007	65 (54–74)
2 doses, strains unspecified	10	0.36 (0.28–0.45)	0.36 (0.28–0.45)	0 (0–69)	.682	64 (55–72)
2 doses, age <1 y	13	0.30 (0.23–0.40)	0.33 (0.27–0.40)	33 (0–65)	.118	70 (60–77)
2 doses, age ≥1 y	13	0.42 (0.29–0.61)	0.44 (0.36–0.54)	70 (46–83)	<.001	58 (39–71)
Sensitivity analyses						
2 doses, excluding referral to ED only or primary healthcare centers	23	0.31 (0.24–0.39)	0.33 (0.29–0.37)	67 (50–79)	<0.001	69 (61–76)
2 doses, excluding HIC countries	16	0.40 (0.33–0.49)	0.39 (0.35–0.45)	48 (7–71)	.016	60 (51–67)
1 dose, excluding HIC countries	9	0.58 (0.50–0.68)	0.58 (0.50–0.68)	0 (0–65)	.488	42 (32–50)
2 doses, excluding unmatched controls or unclear matching process	11	0.24 (0.17–0.34)	0.27 (0.21–0.33)	55 (11–77)	.014	76 (66–83)
1 dose, excluding unmatched controls or unclear matching process	4	0.44 (0.33–0.60)	0.44 (0.33–0.60)	0 (0–85)	.868	56 (40–67)

Abbreviations: CI, confidence interval; ED, emergency department; FE, fixed effect; HIC, high-income country; LMIC, lower-middle-income country; No., number of studies/subgroups included in the analyses; OR, odds ratio; RE, random effect; UMIC, upper-middle-income country; VE, vaccine effectiveness.

^a^Planned analyses for which an insufficient number of articles were identified were not performed (secondary analyses: hospital controls, 2 doses, unmatched/other hospital controls; neighborhood/community controls, 2 doses, unmatched controls; 2 doses, database controls; stratified by SES: 2 doses, low-income countries; 1 dose, HIC; stratified by disease severity: 2 doses, mild severity; 1 dose, mild severity; 1 dose, moderate severity; stratified by strain: all analyses for 1-dose VE; stratified by age: all analyses for 1-dose VE).

^b^Calculated using the RE model.

VE for 2 doses of *Rotarix* was 81% (95% CI, 74% to 86%), 54% (95% CI, 39% to 65%), and 63% (95% CI, 50% to 72%) in high-, upper-middle-, and lower-middle-income countries, respectively. One-dose VE also varied slightly with the SES of the countries, from 48% (95% CI, 33% to 60%) for upper-middle-income to 37% (95% CI, 23% to 48%) for lower-middle-income countries (Table 2). In a stratified analysis by type of control, the pooled 2-dose VE of *Rotarix* varied between 81% (95% CI, 69% to 88%) for matched and 63% (95% CI, 52% to 72%) for unmatched RV test–negative controls (Table 2).

When the analysis was performed by strain type, the estimated 2-dose VE of *Rotarix* was 89% (95% CI, 82% to 93%), 72% (95% CI, 65% to 78%), and 65% (95% CI, 54% to 74%) for the G1P [8] genotype, partially heterotypic strains, and fully heterotypic strains, respectively (Table 2). Stratified analysis by age groups showed higher 2-dose VE point estimates in children aged <1 year (70%; 95% CI, 60% to 77%) than in those ≥1 year of age (58%; 95% CI, 39% to 71%) (Table 2; Supplementary Figure 2). Two-dose VEs for high and very high disease severity were 64% (95% CI, 50% to 74%) and 60% (95% CI, 38% to 74%), respectively; 1-dose VEs for high and very high disease severity were 38% (95% CI, 16% to 54%) and 30% (95% CI, –28% to 62%), respectively.

Stratified analyses on 2 levels performed for the 2-dose VE confirmed a higher VE in children aged <1 year than in those ≥1 year of age, in both upper-middle- and lower-middle-income countries (Supplementary Table 1).

When excluding data from studies conducted only in ED settings or primary health care centers (not in hospital settings), sensitivity analyses yielded a 2-dose VE of 69% (95% CI, 61% to 76%), similar to that obtained from the main analysis. When omitting data from high-income countries, VE estimates were found to be in the same ranges as those obtained for the main analysis (ie, 60%; 95% CI, 51% to 67%; and 42%; 95% CI, 32% to 50%; for 2 doses and 1 dose, respectively). Higher VE estimates for both 2 doses and 1 dose of *Rotarix* were observed when excluding unmatched controls or studies where the matching process was not clear (Table 2).

Funnel plots assessing publication bias are presented in Supplementary Figure 3. Egger’s regression test showed no significant funnel plot asymmetry for the 2-dose VE analysis (calculated coefficient of –1.23; 95% CI, –3.08 to 0.63; *P* = .19), whereas significant asymmetry was shown by the coefficient for the 1-dose VE (–1.46; 95% CI, –3.73 to –0.19; *P* = .03).

## DISCUSSION

This meta-analysis of the VE of the *Rotarix* vaccine includes peer-reviewed data publicly available on its use in real-world settings over a period of 10 years. Although several meta-analyses assessing *Rotarix* effectiveness against various end points have already been published [5, 56–58], we included worldwide data and performed subgroup analyses, thus providing an exhaustive view on the VE of *Rotarix* against RVGE-related hospitalizations or ED visits. Our systematic review and meta-analysis showed that programmatic use of *Rotarix* prevents hospital admission or ED visits due to RVGE in children under 5 years of age.

We analyzed data from 29 studies assessing postlicensure VE, conducted in various geographical regions, and covering the entire range of SES. Most of the studies included in the review were undertaken in countries where *Rotarix* is implemented in the NIP. Studies from Taiwan and Portugal, where the vaccine is only available on the private market, were also included.

The meta-analysis showed that a 2-dose schedule of *Rotarix* provided considerable prevention of RV disease–related hospitalizations or referrals to ED, with a pooled VE estimate of 69%, but increasing to up to 81% in high-income countries. The estimated VE for 1 dose was 46%, indicating that partial reduction of hospitalization is also provided by 1 dose of *Rotarix.* However, our results highlight the importance of a full vaccination schedule.

Overall VE values obtained from stratified analyses were the highest in high-income countries. This is consistent with results from clinical trials, showing lower vaccine efficacy in African countries [11] than in industrialized countries [13]. Lower VE in low-income countries was also noted for other live oral vaccines and has been attributed to differences between countries of different SES in breastfeeding practices, micronutrient malnutrition, gut flora, RV epidemiology, and underlying medical conditions [59].

Because case-control study design is a widely used method to assess VE, only this type of study was included in our meta-analysis. In addition, to ensure high specificity of the outcome and avoid any potential misclassification, only studies with laboratory-confirmed RVGE diagnosis were considered when conducting the meta-analysis. The type of controls used was previously shown to impact VE to some extent, as observed in a review performed to assess VE of RV vaccines in Latin America [56]. In our study, we considered all types of controls, in line with other meta-analyses performed for RV vaccines [5, 56, 57] and selected the control groups according to a prespecified selection method. Only peer-reviewed studies that were checked for quality before inclusion in the meta-analysis were used; therefore, an adequate homogeneity between cases and controls in terms of exposure to the disease was assumed. Of the selected studies, when available, we preferentially included in the meta-analysis those reporting estimates based on hospital controls over community controls and studies using matched controls to limit bias due to any potential confounders. To assess the choice of controls on the overall VE estimate, we also performed subgroup analyses by type of control. Similar to previous meta-analyses [56], we evidenced a relationship between VE and the type of controls used, as studies using hospital matched controls yielded higher VE of the 2-dose schedule of *Rotarix* than those with unmatched or neighborhood/community controls, with values varying from 81% to 63%. Furthermore, RV test–negative control types are highly specific for non-RV diseases and thereby increase the robustness of the estimate. The overall VE of 2 *Rotarix* doses obtained using matched RV test–negative controls was 81%, but only 4 studies could be included in the analysis.

Vaccine efficacy as assessed in clinical trials was previously shown to increase with the severity of the disease [13], a finding partially confirmed in real-world settings [57]. In our analysis, *Rotarix* VE was similar against disease of high and very high severity regardless of the number of doses but could not be estimated against mild and moderately severe disease due to the lack of data.

VE against RVGE-related hospitalizations or ED visits varied between 64% and 89% with the type of RV strain, in line with efficacy results observed in clinical trials that demonstrated broad protection against severe RVGE by different RV types [10, 14]. The highest VE estimates were found for homotypic strains and confirm the high protective effect of Rotarix against fully homotypic strains. A higher point estimate for VE was observed in children aged <1 year compared with children aged ≥1 year. Nevertheless, lower odds of RV-related hospitalizations or referrals to ED were still observed in our study in vaccinated vs unvaccinated children ≥1 year of age.

A rigorous quality control procedure has been applied for both the systematic review and the meta-analysis. Additional analyses investigating the publication bias have been performed. The study’s main strength was the inclusion of robust case-control studies using laboratory confirmation of RV status. To minimize the risk of bias, adjusted results were preferentially included in the analysis. Nevertheless, although the case-control design allowed for stratified meta-analyses performed by type of control, this type of study also presents a certain risk of bias, as it relies on retrospectively assessed chart-based data and thorough documentation of vaccination history that might not have been correctly reported.

The study also has several limitations. Although covering various geographical settings, the data used in the meta-analysis originated from only 21 countries, with a small amount of data from low-income countries. Similarly, a relatively small number of studies reported strain-specific data. For certain subgroups, only a limited number of studies could be included in the analyses, or no meta-analyses could be performed due to insufficient data. The funnel plots suggest publication bias for some small studies with high OR (or low VE). However, as these studies had very low weights, their impact on the overall estimates is low.

Importantly, as previously discussed in relation with postlicensure studies [60], values derived from case-control studies can only provide information on the direct effectiveness of a vaccine and do not fully account for indirect protection afforded by herd effect, following the implementation of a vaccination program [60]. Given that a disease reduction of up to 75% was estimated in age groups that were not vaccine-eligible in countries with national RV immunization programs [61–63], VE values from our study are likely to underestimate the true global impact of *Rotarix* vaccination.

## CONCLUSIONS

This meta-analysis provides strong evidence that vaccination with 2 doses of *Rotarix* has a substantial preventive effect against hospitalizations and ED visits due to RVGE, further confirming the effectiveness of *Rotarix* in children younger than age 5 years across various geographic and economic settings.

## Supplementary Data

Supplementary materials are available at *Open Forum Infectious Diseases* online. Consisting of data provided by the authors to benefit the reader, the posted materials are not copyedited and are the sole responsibility of the authors, so questions or comments should be addressed to the corresponding author.

Supplementary Data
