# Supplementary material for: Effectiveness of the Oral Human Attenuated Rotavirus Vaccine: A Systematic Review and Meta-analysis—2006–2016
Source: Open Forum Infect Dis. 2018 Nov 12;5(11):ofy292. doi: 10.1093/ofid/ofy292 (PMC6284461; doi:10.1093/ofid/ofy292)
Supplement: Supplementary Data [file ofy292_suppl_supplementary_data.docx]

**Supplementary text 1.** Search strategy of peer-reviewed articles

Search string for PubMed

"RotaTeq"[Supplementary Concept] OR RotaTeq[tiab] OR RV5[tiab] OR "RIX4414 vaccine" [Supplementary Concept] OR RIX4414[tiab] OR Rotarix[tiab] OR RV1[tiab] OR "Rotavirus Vaccines"[Mesh] OR rotavirus vaccin*[tiab] OR (("Rotavirus"[Mesh] OR "Rotavirus Infections"[Mesh] OR rotavir*[tiab]) AND ("Vaccination"[Mesh] OR vaccin*[tiab] OR "Immunization"[Mesh] OR immun*[tiab]))

Search string for Cochrane

RotaTeq OR RV5 OR RIX4414 OR Rotarix OR RV1 OR (rotavirus vaccin*)

Limits

Publication date: 01 January 2006 – 07 July 2016

Number of hits

A first search (conducted on 2 April 2015) yielded 2506 hits. A second search, covering the period April 2015 to 7 July 2016, using the same strings was performed on 7 July 2016 to update the literature review, and the analysis yielded another 480 results.

**Supplementary text 2.** Inclusion/exclusion criteria for the systematic literature search and meta-analysis

**A. Systematic literature search**

*Inclusion criteria:*

- Data relevant for the objectives (to describe the effectiveness of *Rotarix*)
- Post-licensure studies (e.g. cohort, case-control, or before-after studies)
- Original study that provided data on vaccine effectiveness

*Exclusion criteria:*

- Clinical trials
- Efficacy studies
- Non-pertinent publication types (e.g. letters to the editor, editorials or comments)
- Genetic studies, biochemistry or molecular studies
- Treatment studies
- Studies on vaccination coverage
- Studies focusing only on post-licensure genotype distribution
- Studies about laboratory methods
- Animal studies
- Modelling studies
- Economic evaluations
- A narrative review (e.g. no methods section that describes the way the authors collected the literature)
- Methods section did not provide sufficient details to understand what has been done
- No quantitative data could be retrieved from the article
- No full-text available
- Meta-analysis or systematic reviews (the reference list was checked for any other relevant articles)
- Results not stratified by vaccine brand (i.e. results are based on both *Rotarix* and *RotaTeq* patients)
- Vaccine brand not reported (i.e. in the whole study there was no indication for one of the rotavirus vaccines)
- No study endpoint of interest (i.e. cases with intussusception instead of rotavirus)
- Study does not provide relevant data for the objectives (e.g.: indirect protection of individuals in households whose children had received rotavirus vaccine versus children that did not receive rotavirus vaccine; only pre- or post-licensure data reported; results based on previously published studies).

**B. Meta-analysis**

*General inclusion criteria:*

- Observational studies (no clinical trial);
- Studies assessing direct vaccine effectiveness (no impact study);
- Pediatric studies including children until 5 years of age;
- Birth cohort eligible for rotavirus (RV) vaccination;
- Endpoints related to hospitalization or emergency department (ED) visits for RV.

*Rotavirus diseases inclusion criteria*

- Outcome of study is (vaccination status of) RV-gastroenteritis hospitalization or ED visits;
- RV diseases confirmed by laboratory testing.

**Supplementary Figure 1.** Estimated pooled vaccine effectiveness for one dose of *Rotarix* against laboratory-confirmed rotavirus infection after hospital and/or emergency department visits


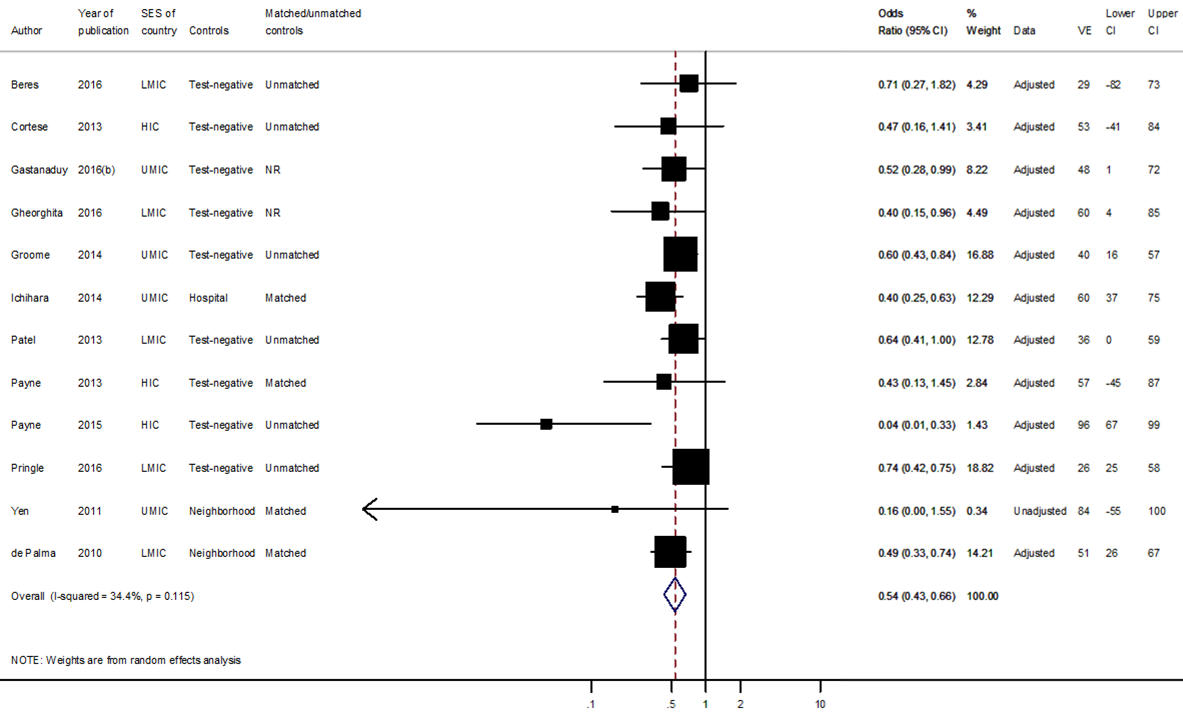


CI, confidence interval; HIC, high-income country; LMIC, lower middle-income country; NR, not reported; SES, socio-economic status; UMIC, upper middle-income country; VE, vaccine effectiveness.

**Supplementary Figure 2.** Estimated pooled vaccine effectiveness for two doses of *Rotarix* against laboratory-confirmed rotavirus infection after hospital and/or emergency department visits, by age


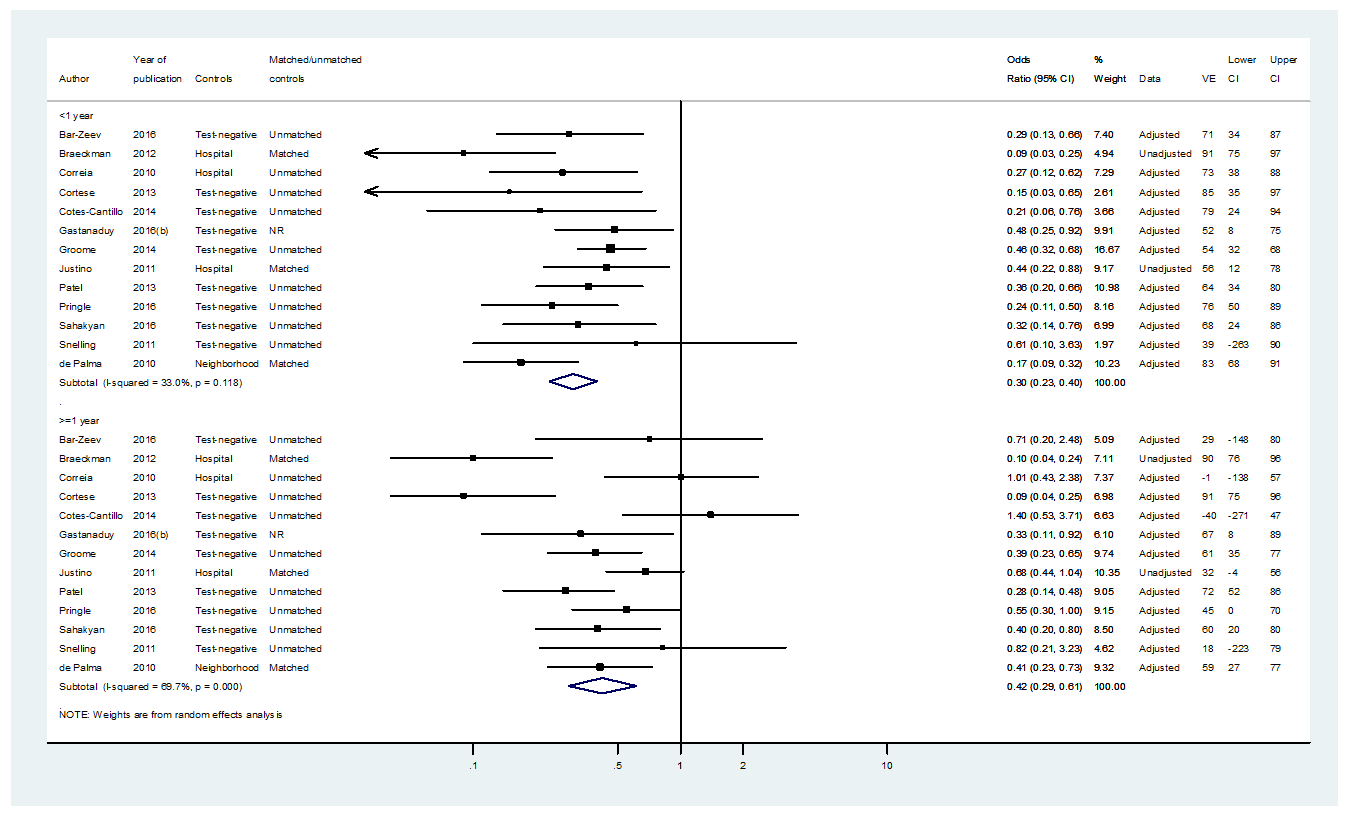

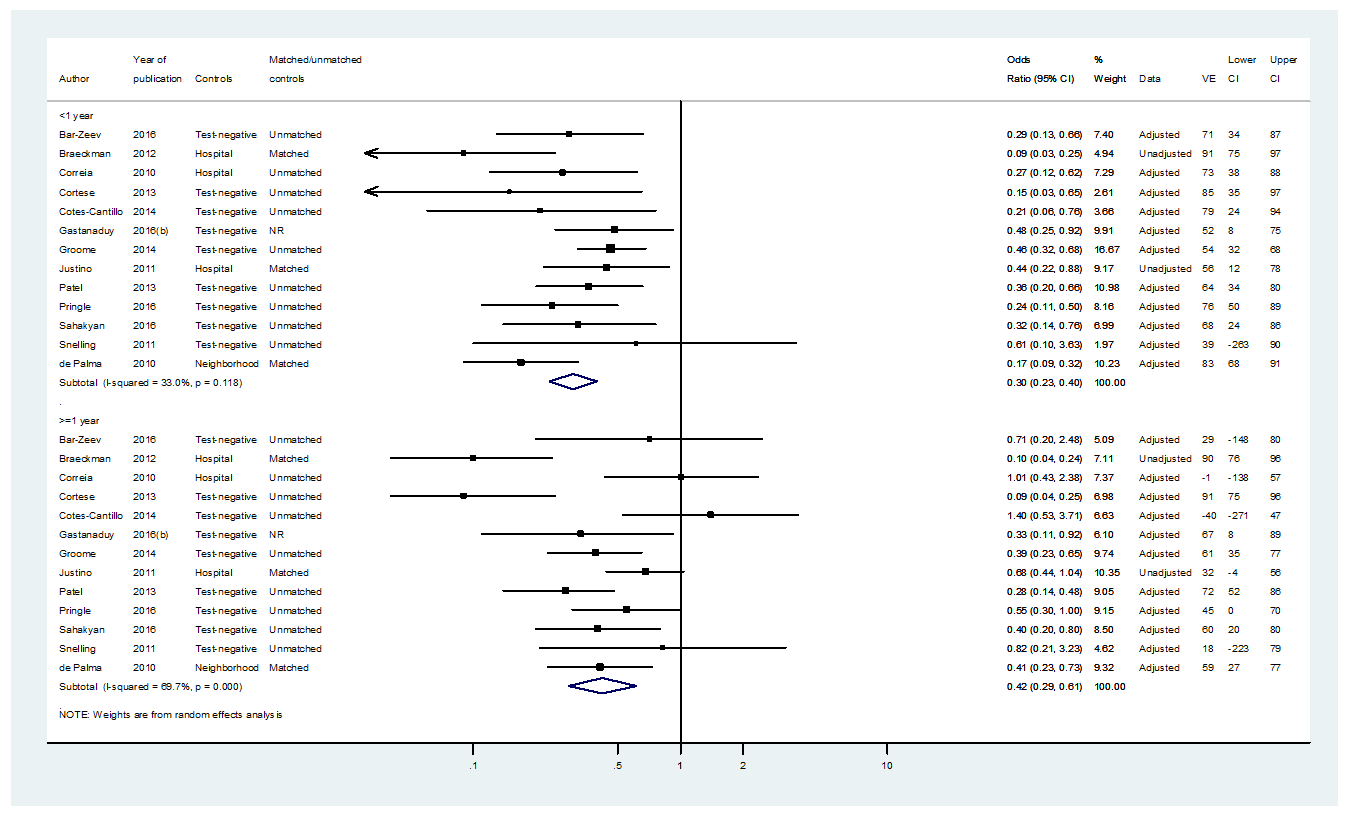

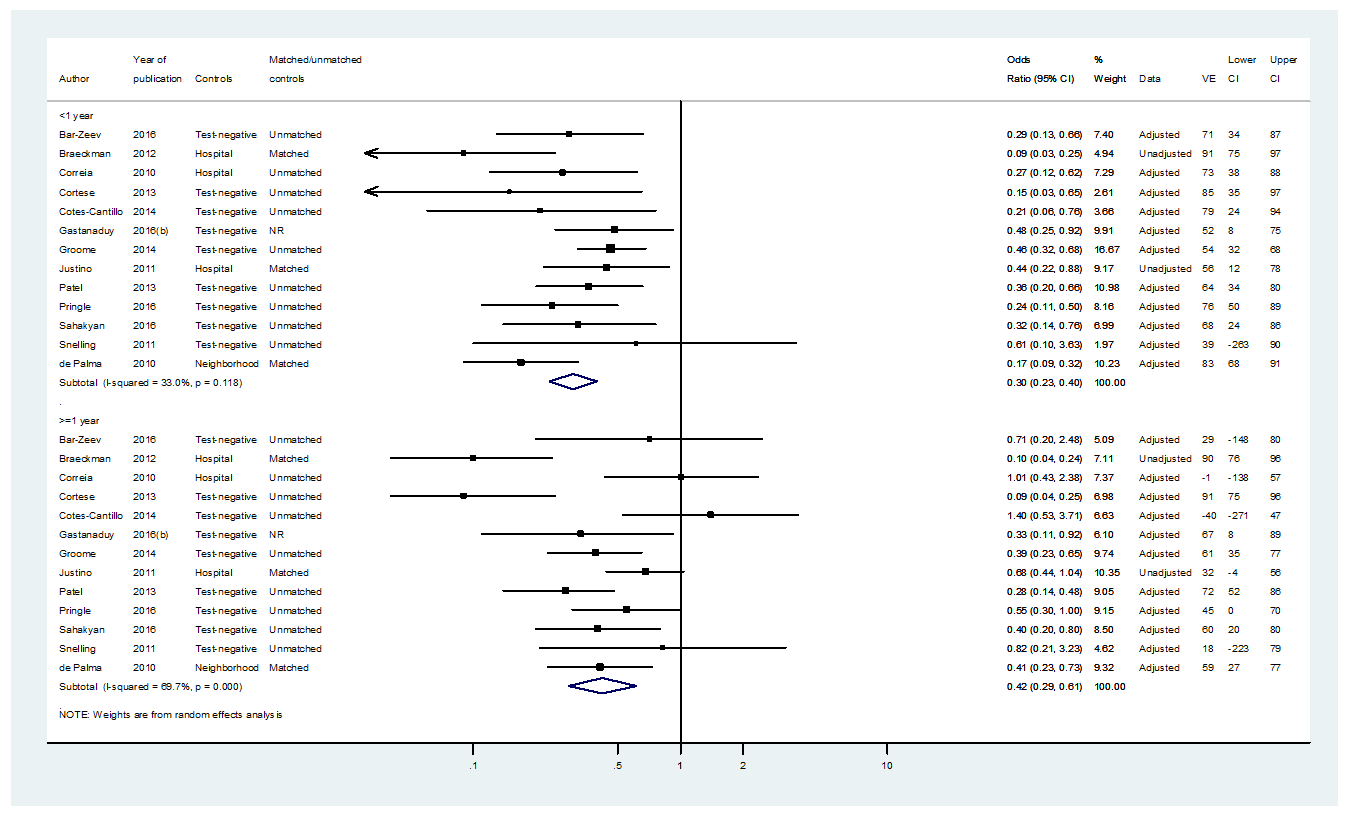

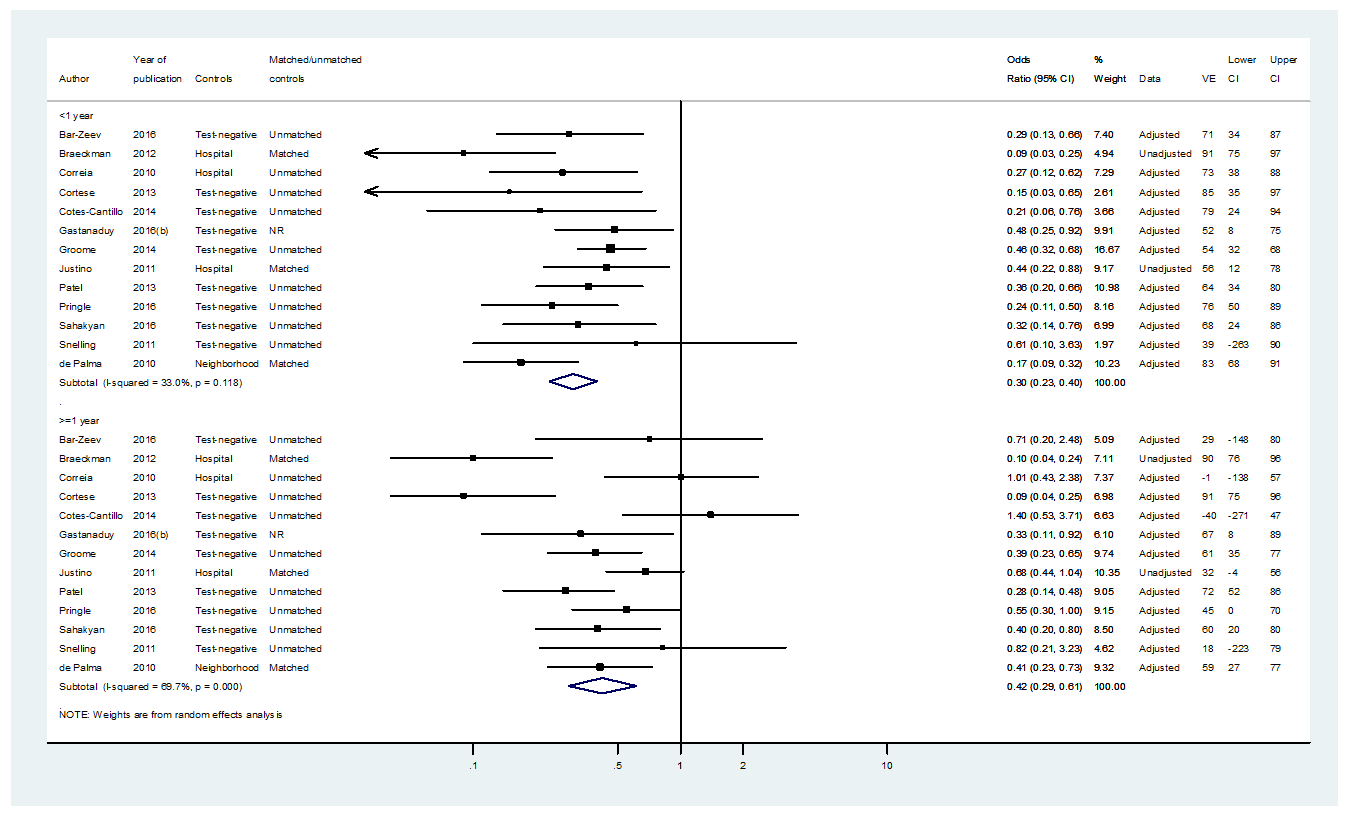

CI, confidence interval; NR, not reported; VE, vaccine effectiveness.

**Supplementary Figure 3.** Assessment of publication bias for 2- (A) and one-dose (B) VE of *Rotarix* against laboratory-confirmed rotavirus infection after in hospital- and/or ED –emergency department visits


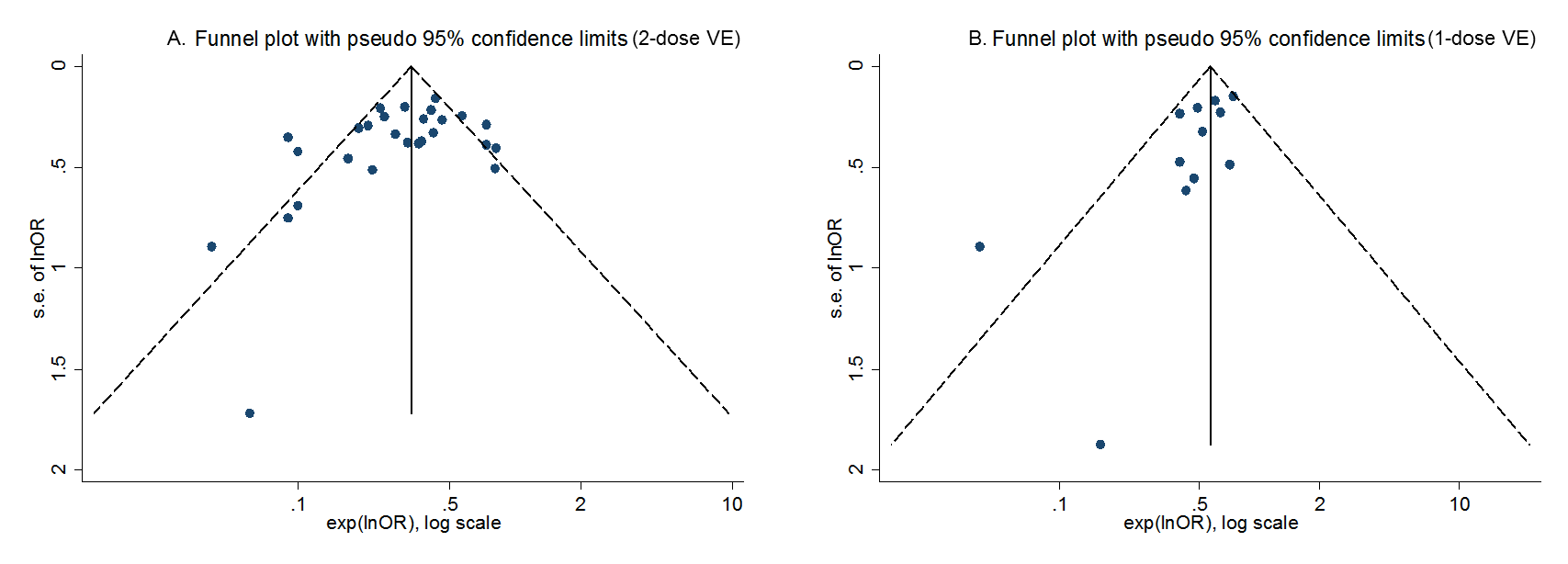


OR, odds ratio; s.e., standard error; VE, vaccine effectiveness.

**Supplementary Table 1.** Overview of overall odds ratios and vaccine effectiveness against laboratory-confirmed rotavirus infection after hospital and/or emergency department visits for *Rotarix* vaccination, resulting from 2-level stratified meta-analysis^a^

| Analyses | | N | OR (95%CI) | | | |  | I^2^ (95%CI) | p-value  (Cochrane Q-test) | VE (95%CI)^b^ |
| --- | --- | --- | --- | --- | --- | --- | --- | --- | --- | --- |
|  |  |  | RE model | | | FE model |  |  |  |  |
| *Analyses stratified by age and SES* |  | | |  |  |  |  |  |  |  |
| 2 doses age <1 year and LMIC | | 4 | 0.26 (0.18–0.37) | | | 0.26 (0.18–0.37) |  | 5 (0–85) | 0.371 | 74 (63–82) |
| 2 doses age <1 year and UMIC | | 5 | 0.42 (0.32–0.55) | | | 0.42 (0.32–0.55) |  | 0 (0–79) | 0.616 | 58 (45–68) |
| 2 doses age ≥1 year and LMIC | | 4 | 0.40 (0.29–0.55) | | | 0.40 (0.29–0.55) |  | 0 (0–85) | 0.500 | 60 (45–71) |
| 2 doses age ≥1 year and UMIC | | 5 | 0.63 (0.40–1.00) | | | 0.61 (0.46–0.81) |  | 53 (0–83) | 0.074 | 37 (0–60) |
| *Analyses stratified by strain and SES* | |  |  | | |  |  |  |  |  |
| 2 doses fully heterotypic strains and LMIC | | 2^c^ | 0.39 (0.29–0.53) | | | 0.39 (0.29–0.53) |  | 0 (0–79) | 0.844 | 61 (47–71) |
| 2 doses fully heterotypic strains and UMIC | | 5 | 0.40 (0.24–0.64) | | | 0.41 (0.32–0.52) |  | 68 (18–88) | 0.013 | 60 (36–76) |

CI, confidence interval; FE, fixed effect; LMIC, lower middle-income country; N, number of studies included in the analyses; OR, odds ratio; RE, random effect; SES, socio-economic status; UMIC, upper middle-income country; VE, vaccine effectiveness.

Note: ^a^Analyses for the other combinations of levels and for one-dose VE were planned, but not performed, due to the insufficient number of subgroups (stratified by age and SES: 2 doses age <1 year and low-income countries [LIC], 2 doses age <1 year and high-income countries [HIC], 2 doses age ≥1 year and LIC; 2 doses age ≥1 year and HIC; all analyses for one dose VE; Stratified by strain and socioeconomic level: 2 doses homotypic strain and LIC; 2 doses homotypic strain and LMIC; 2 doses homotypic strain and UMIC; 2 doses homotypic strain and HIC; 2 doses partly heterotypic strain and LIC; 2 doses partly heterotypic strain and LMIC; 2 doses partly heterotypic strain and UMIC; 2 doses partly heterotypic strain and HIC; 2 doses fully heterotypic strains and LIC; 2 doses fully heterotypic strains and HIC; 2 doses strains unspecified and LIC; 2 doses strains unspecified and LMIC; 2 doses strains unspecified and UMIC; 2 doses strains unspecified and HIC; all analyses for one-dose VE).

^b^Calculated using the RE model.

^c^Five subgroups were included.
